# Supplementary figures and images for: The Autophagy Inhibitor Bafilomycin Inhibits Antibody-Dependent Natural Killer Cell-Mediated Killing of Breast Carcinoma Cells
Source: Int J Mol Sci. 2025 Jun 28;26(13):6273. doi: 10.3390/ijms26136273 (PMC12250358; doi:10.3390/ijms26136273)

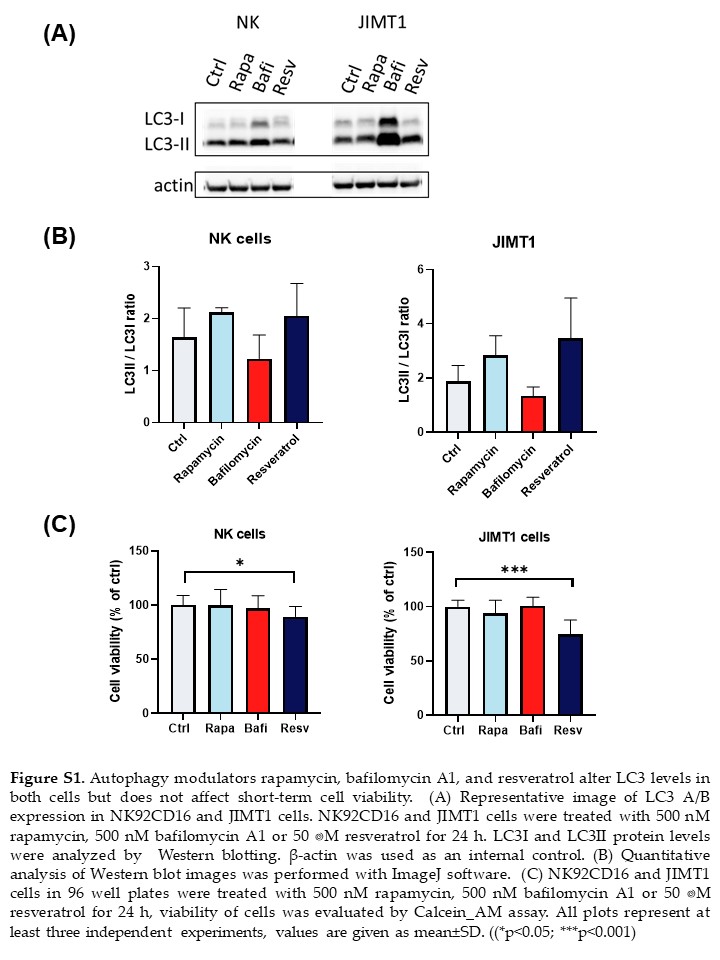

Supplement: Supplementary file 1 [file ijms-26-06273-s001.zip › ijms-3529309-supplementary.jpg]
